# Telomere Recombination Accelerates Cellular Aging in *Saccharomyces cerevisiae*

**DOI:** 10.1371/journal.pgen.1000535

**Published:** 2009-06-26

**Authors:** Xiao-Fen Chen, Fei-Long Meng, Jin-Qiu Zhou

**Affiliations:** The State Key Laboratory of Molecular Biology, Institute of Biochemistry and Cell Biology, Shanghai Institutes for Biological Sciences, Graduate School of the Chinese Academy of Sciences, Chinese Academy of Sciences, Shanghai, People's Republic of China; Fred Hutchinson Cancer Research Center, United States of America

## Abstract

Telomeres are nucleoprotein structures located at the linear ends of eukaryotic chromosomes. Telomere integrity is required for cell proliferation and survival. Although the vast majority of eukaryotic species use telomerase as a primary means for telomere maintenance, a few species can use recombination or retrotransposon-mediated maintenance pathways. Since *Saccharomyces cerevisiae* can use both telomerase and recombination to replicate telomeres, budding yeast provides a useful system with which to examine the evolutionary advantages of telomerase and recombination in preserving an organism or cell under natural selection. In this study, we examined the life span in telomerase-null, post-senescent type II survivors that have employed homologous recombination to replicate their telomeres. Type II recombination survivors stably maintained chromosomal integrity but exhibited a significantly reduced replicative life span. Normal patterns of cell morphology at the end of a replicative life span and aging-dependent sterility were observed in telomerase-null type II survivors, suggesting the type II survivors aged prematurely in a manner that is phenotypically consistent with that of wild-type senescent cells. The shortened life span of type II survivors was extended by calorie restriction or *TOR1* deletion, but not by Fob1p inactivation or Sir2p over-expression. Intriguingly, rDNA recombination was decreased in type II survivors, indicating that the premature aging of type II survivors was not caused by an increase in extra-chromosomal rDNA circle accumulation. Reintroduction of telomerase activity immediately restored the replicative life span of type II survivors despite their heterogeneous telomeres. These results suggest that telomere recombination accelerates cellular aging in telomerase-null type II survivors and that telomerase is likely a superior telomere maintenance pathway in sustaining yeast replicative life span.

## Author Summary

Telomeres are the specialized structures at the ends of eukaryotic linear chromosomes. The simple guanine-rich DNA repeats at telomeres and their associated proteins are important for chromosome stability. Most eukaryotic species have evolved an enzyme named telomerase to replicate their telomeric DNA. Telomerase usually contains a protein catalytic subunit and a RNA template subunit. A few eukaryotic species can use either telomere recombination or retrotransposon-mediated transposition to accomplish telomere elongation. Interestingly, the baker's yeast *Saccharomyces cerevisiae* can use both telomerase and recombination to replicate telomeres. In this study, we utilize this unique eukaryotic model system to compare the efficiency of these two mechanisms in the maintenance of cellular function and life span. Telomerase-null cells that used recombination to elongate telomeres were able to maintain relatively stable chromosomes; however, they exhibited a shortened replicative life span which may represent a novel aging pathway. Reintroduction of telomerase inhibited telomere recombination and restored the replicative life span of these cells, implying that telomerase is superior to telomere recombination in the regulation of yeast replicative life span.

## Introduction

Telomeres are the physical ends of linear eukaryotic chromosomes and are composed of specific repetitive DNA sequences and binding proteins [1],[2]. The functional integrity of telomeres is required for cell proliferation and survival because they protect chromosome ends from nucleolytic degradation and help to distinguish normal chromosome ends from DNA double-strand breaks [3]–[5]. Additionally, telomeres can compensate for the incomplete replication of chromosomal DNA by conventional DNA polymerases [6],[7].

In eukaryotes, telomeres can be maintained by three different mechanisms, namely a telomerase-dependent pathway, a recombination pathway and a retrotransposon-mediated transposition pathway [8],[9]. Telomerase-dependent telomere replication has been documented in the vast majority of eukaryotic species including in budding yeast and humans. In these species, repetitive telomeric DNA is added to the chromosome ends by telomerase, a specialized reverse transcriptase that catalyzes the addition of telomeric DNA using its intrinsic RNA template [10],[11]. Recombination-dependent telomere maintenance has been reported in a few organisms that naturally lack telomerase, including the lower dipterans *Chironomus* and *Anopheles* and the plant *Allium spp* [12]–[14]. Retrotransposon-mediated telomere maintenance has been well adapted by the fruit fly *Drosophila melanogaster* [15]–[18]. The wider use of telomerase in eukaryotes suggests that it has been evolutionarily selected for as an advantageous mechanism for maintaining telomere integrity and stability, however, the reasons why telomerase has been adopted by so many eukaryotic species are not clear.

Interestingly, some organisms are likely capable of using both telomerase and recombination to replicate their telomeres. For example, previous studies have reported that 85% of the human cancer cells are telomerase positive, however the other 15% cancer cells are telomerase negative [19] and maintain their telomeres by recombination pathway, also termed alternative lengthening of telomeres (ALT) [20]. In the budding yeast *Saccharomyces cerevisiae*, a *RAD52*-dependent homologous recombination pathway can be employed by a minority of telomerase-negative cells as an alternative method for telomere maintenance. These cells are called post-senescent survivors. Two types of post-senescent survivors exist and are distinguishable by their characteristic telomere patterns. Type I survivors exhibit amplification of Y' elements and have very short TG_1–3_ repetitive tracts on the chromosome ends [21]. Type II survivors show a variable pattern of long tracts of TG_1–3_ repeats and only modest Y' amplification [22]. Because type II survivors have long and heterogeneous telomeric repeats, and their telomere maintenance requires both *RAD50* and *RAD52*, they resemble human ALT cells [20], [23]–[25]. In budding yeast, telomerase seems to be the preferential telomere-elongation pathway. Introduction of the telomerase component *EST1* back into an *est1Δ* type I survivor that exhibits extensive Y' amplification results in the elongation of the terminal telomeric tract back to wild-type length, as well as a substantial reduction in Y' copy number [21],[22]. Similarly, following reintroduction of telomerase into a type II survivor, telomeres gradually return to a wild-type telomeric structure that can be confirmed by examining telomeric restriction patterns via Southern blotting [22].

To investigate whether recombination is inferior to telomerase in preserving an organism or cell under natural selection, we compared cellular traits in telomerase-null post-senescent type II survivor cells to cellular traits in telomerase positive cells. Type I recombination survivors were not included in this report because they have a severe growth defect and highly abnormal karyotypes [21],[26]. In this report we demonstrate that recombination was as efficient as telomerase in maintaining cell survival and overall genome stability, but telomerase-null cells using recombination-only maintenance of telomeres had a shortened replicative life span (RLS) when compared to telomerase-positive cells. In yeast, RLS is defined as the total number of daughter cells generated by a mother cell before cell death [27]. RLS was significantly reduced in type II survivors. The decline in RLS was not due to a defect in the canonical aging regulation pathways and the reintroduction of telomerase activity immediately restored the RLS of type II survivors to that of a wild type cell. Our results provide experimental evidence supporting the notion that telomerase is superior to telomere recombination in the regulation of yeast replicative life span.

## Results

### Telomere recombination is as efficient as telomerase in maintaining cell survival

**Figure 1:**
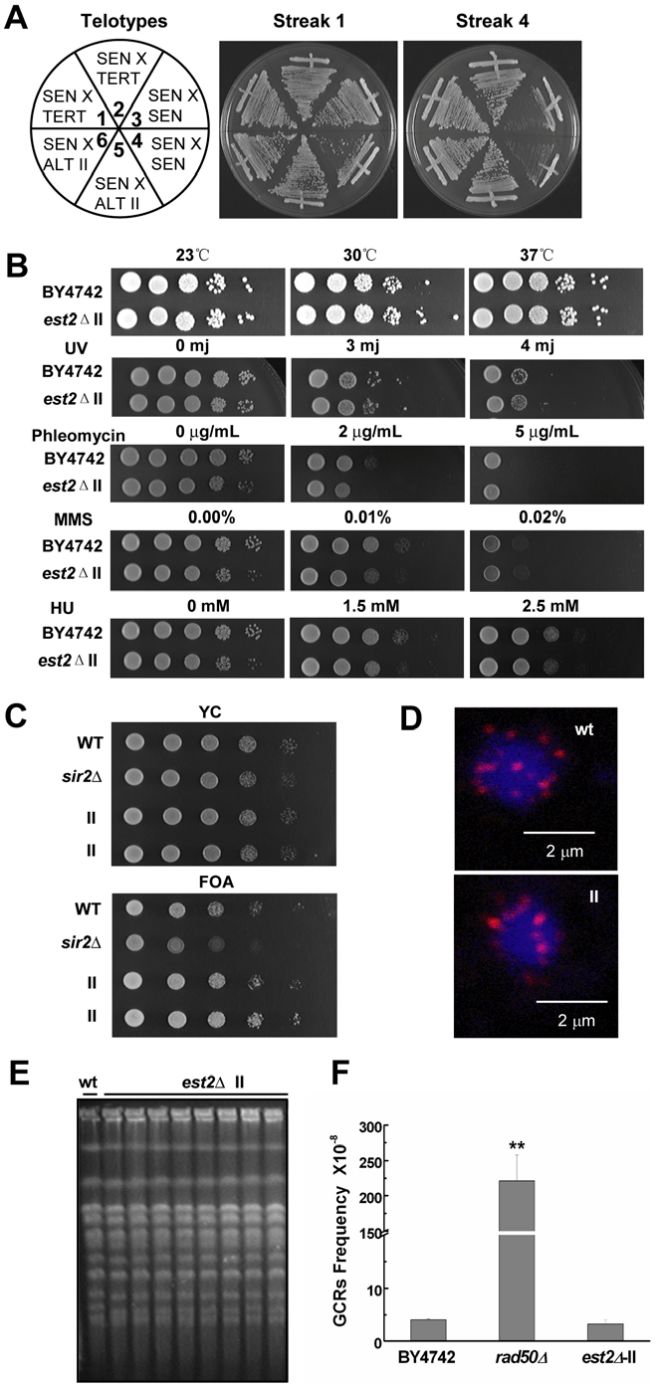
Phenotypic analyses of BY4742 *est2Δ* type II survivors. (A) Cell growth of diploids derived from crosses between: SEN *est2Δ* cells and SEN *est2Δ* cells (sectors 3, 4, negative control for senescence), SEN *est2Δ* cells and TERT telomerase positive cells (sectors 1, 2), SEN *est2Δ* cells and ALT II *est2Δ* strains (sectors 5, 6). (B) DNA damage sensitivity assay with *est2Δ* type II survivors. (C) Telomere position effect assay of *est2Δ* type II survivors. The *sir2Δ* strain serves as a positive control. (D) Immunostaining of the telomeric repeat binding protein Rap1p (Red) in *est2Δ* type II survivors. Blue, DAPI staining of DNA. (E) Pulsed-field gel electrophoresis (PFGE) analysis of karyotypes in *est2Δ* type II survivors. Each lane represents an independent clone. (F) Gross chromosomal rearrangement (GCR) analysis. Mean and standard deviation (S.D.) rates collected from three independent experiments are shown. The *rad50Δ* strain serves as a positive control for increased GCRs.

To determine whether the recombination pathway is as efficient as the telomerase pathway in maintaining cell survival, we mated either type II survivors (ALT II pathway in humans) or telomerase-proficient cells (TERT pathway in humans) with yeast cells whose telomerase was recently inactivated (pre-survivors, SEN) (Figure 1A). The viability of the resulting diploids was examined. Diploids generated by crossing two SEN populations senesced and underwent crisis (Figure 1A, sectors 3 and 4). In contrast, diploids generated by crossing a type II survivor with a SEN (Figure 1A, sectors 5 and 6) grew as vigorously as the diploids created by mating a TERT with a SEN (Figure 1A, sectors 1 and 2). These data are consistent with previous reports [28] and suggest that telomere maintenance by recombination is as efficient as telomere maintenance by telomerase in maintaining cell survival.

### Telomerase-null type II survivors are able to stably maintain their chromosomes

Genome integrity is maintained in telomerase-proficient cells because telomeres are not recognized as DNA double-strand breaks [3]. To ascertain if genome stability was altered in telomerase-negative type II survivors, we carried out several phenotypic analyses and compared our results to those obtained from telomerase-positive (wild-type) cells. First, we determined that type II survivor cells grew as robustly as the wild-type cells (Figure 1B) [22]. Second, we found that type II survivors exhibited similar levels of sensitivity to four separate DNA damage-inducing agents when compared to wild-type cells (Figure 1B). Third, telomere position effect (TPE), a silencing mechanism combining telomere architecture and classical heterochromatin features, was slightly enhanced in type II survivor cells, indicating that the heterochromatic state of telomeres has not been damaged (Figure 1C). This observation was consistent with previous reports where increasing the length of telomeres was found to enhance TPE [29]–[31]. Fourth, normal telomere positioning at the nuclear periphery was maintained in type II survivors, as shown by immunostaining of the telomeric repeat binding protein Rap1p (Figure 1D). Fifth, pulsed-field gel electrophoresis revealed that haploid type II survivors contained linear chromosomes that were indistinguishable from wild-type cells (Figure 1E). Finally, the gross-chromosomal rearrangement (GCR) analyses showed that the type II survivors had a very low GCR rate, in contrast with the *rad50Δ* mutants that displayed a significant increase of GCR events (Figure 1F). This behavior of the type II survivors was similar to that seen in wild-type cells as previously reported (Figure 1F) [32]. These results suggested that the elevated recombination at type II survivor telomeres has not caused any noticeable defects in DNA replication or repair, and the chromosomes of the type II survivors are stably maintained.

### Telomerase-null type II survivors age prematurely

**Figure 2:**
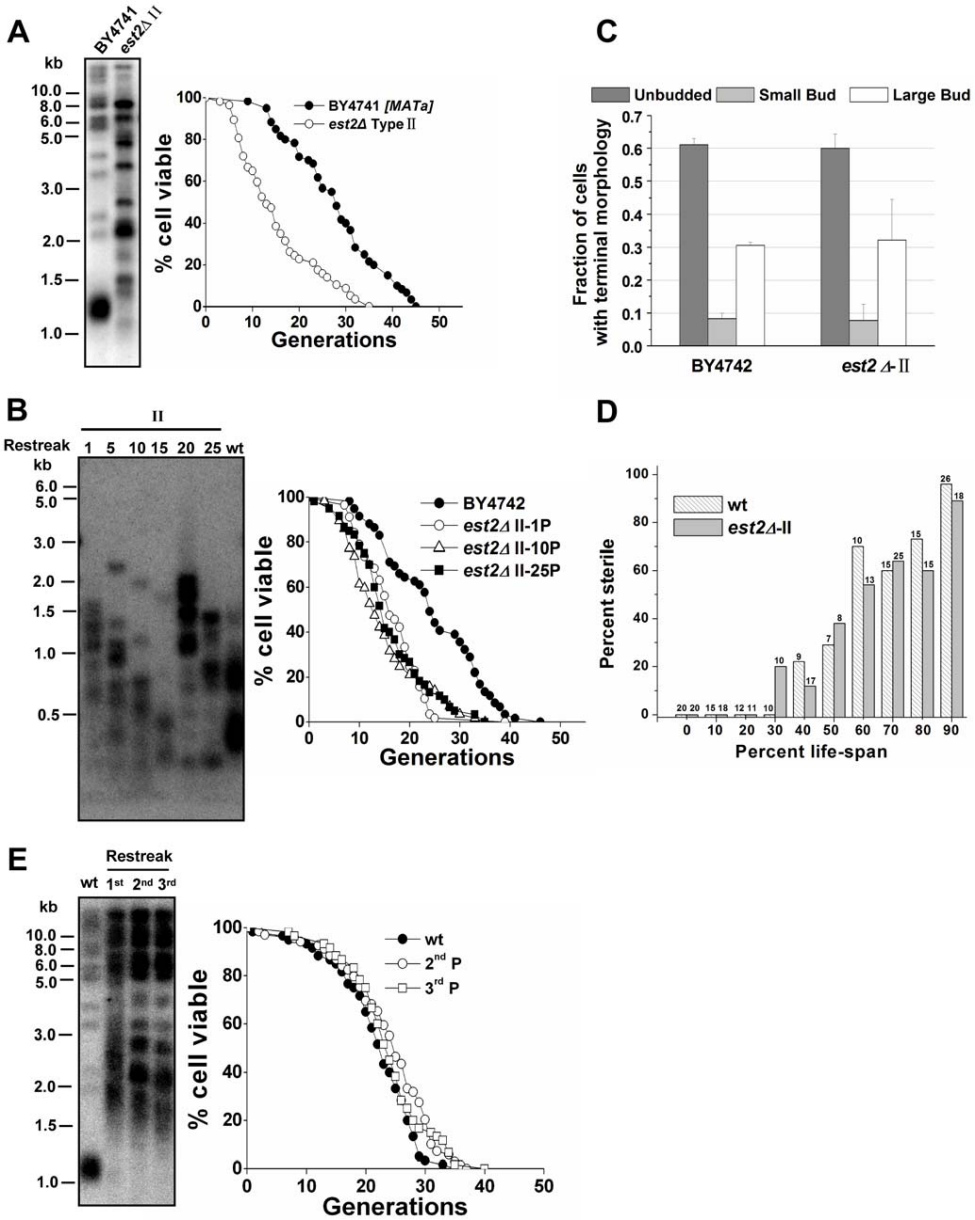
Premature aging of telomerase-null type II survivors. (A) Telomere analysis (left) and life span assay (right) of telomerase-null type II survivors. The number of daughter cells (generations) produced per mother cell are plotted as a function of mother cell viability. Average life span of strains shown (sample size): BY4741, 27.8±9.7 (n = 60); *est2Δ*-type II (*MATa*), 15.3±8.5 (n = 57). (B) Changes of telomeres (left) and life span (right) in type II survivors during the outgrowth. Type II survivors were serially restreaked 25 times on YPD plates. (Left) Genomic DNA from the 1^st^, 5^th^, 10^th^, 15^th^, 20^th^, and 25^th^ restreaks was digested with 4 bp cutter (*Msp*I, *Hae*III, *Hinf*I, *Alu*I) for the Southern blot analysis. (Right) Life span of the 1^st^, 10^th^, and 25^th^ restreaks was determined. Average life span of strains shown: BY4742, 24.8±9.6 (n = 59); *est2Δ*-type II -1^st^ P, 16.5±6.2 (n = 57); *est2Δ*-type II -10^th^ P, 15.0±7.9 (n = 57); *est2Δ*-type II -25^th^ P, 16.1±7.6 (n = 60). (C) Terminal morphology of senescent cells. Cells at the end of life span experiments were classified according to the budding pattern as described [37]. Average and S.D. values from three independent experiments are shown (n>50 for each strain in each experiment). (D) α factor responsiveness in old cells. Cells of various ages were scored for their ability to undergo cell cycle arrest and schmooing in response to the yeast mating pheromone, α factor. The number of cells in each data set for each age group is shown above the bar and data is presented as the percentage of cells that did not schmoo in the presence of α factor. (E) Life span (right) and telomere length (left) analysis. Four telomerase components: Est1p, Est2p, Est3p, and *TLC1* RNA were co-overexpressed in BCY123 under the control of GAL1 promoter (Galactose induction). Then cells were serially restreaked on YPD to shut off the over-expression of telomerase. (Left) Telomere blot analysis of the 1^st^, 2^nd^, and 3^rd^ passaged cells. Genomic DNA was digested with *Xho*I. (Right) Life span analysis of the 2^nd^ and 3^rd^ passaged cells. Average life span of strains shown: wild-type, 21.8±6.7 (n = 60); 2^nd^ P, 23.9±7.5 (n = 60); 3^rd^ P, 23.8±6.6 (n = 60).

A link between an increase of repetitive rDNA recombination and cellular aging has been well established in *S.cerevisiae* [33]. With the activation of homologous recombination at telomeric repeats, the type II survivor cells showed more heterogeneous lengths of telomeric TG_1–3_ tracts and modest Y' telomere amplification (Figure 2A) [22]. Conversely, DNA instability was not observed in another part of the genome when gross chromosomal rearrangements were examined in type II survivors (Figure 1F). This discontinuity of results led us to wonder if the replicative capacity of the type II survivors was comparable to that of telomerase-positive cells. A population of budding yeast cells, can be grown indefinitely in culture under optimal conditions using either telomerase or recombination for telomere maintenance (Figure 1A) [21],[22]. However, the replicative capacity of a single yeast cell is finite. This is because of the activity of other functional aging pathways, and this finite life span holds true whether the telomeres are maintained by telomerase or by recombination throughout the life span [33],[34]. Since yeast cells reproduce by asymmetric cell division with a larger mother cell giving rise to a smaller daughter cell, the two cells can be separated right after each cell division by micromanipulation. The number of daughter cells that a mother cell can produce before senescence defines the replicative life span of that cell. The average number of cell divisions undergone by a group of mother cells defines the mean replicative life span of a yeast strain [27],[35]. A young cell will become mother cell after production of its first daughter.

Replicative life span (RLS) analysis showed that the mean life span of the type II survivors was 15.3 generations, which was much shorter than 27.8 generations of wild-type cells (Figure 2A). Apparently, the reduction in the replicative capacity of type II survivors was neither mating-type nor strain-specific (Figure S1A, S1B). Consistently, the type II survivor cells derived from deletion of the telomerase subunit *EST3* also showed significantly decreased life span (Figure S1C). Previous studies showed that, type II survivors are stable over time, but their telomeres experience a cycle of continuous shortening and abrupt elongation during the outgrowth (Figure 2B) [22]. To determine whether the accelerated cellular senescence persists during the outgrowth, we analyzed the life span of the 1^st^-, 10^th^-, and 25^th^-restreaked type II survivors. All these survivors exhibited significantly reduced life span, despite the changes in telomere length (Figure 2B). Thus, for the first time, a severely reduced life span was observed in the telomerase-null type II survivors.

As previously reported, critically short telomere(s) cause telomerase-deficient cells to abruptly cease cell division, and senesce at the G2/M checkpoint (Figure S2) [36]. To determine whether the accelerated senescence of type II survivors was caused by critically short telomere(s), the morphology of cells at the end of their life span was indexed according to a previously established method where the fraction of unbudded, small-budded, and large-budded cells is determined [37]. In our experiments, the large-budded proportion of old type II cells was comparable to that of old wild-type cells (Figure 2C), suggesting that type II survivors may age by a process that is independent of critically-short-telomeres.

Long telomeres have also been proposed to affect replicative life span. For example, *rif1Δ* cells have much longer telomeres than wild-type cells and their replicative life span is reduced [38]. These data with *rif1Δ* cells led to the generation of a hypothesis that long telomeres may reduce life span by competing for Sir silencing factors with the non-telomeric loci [38],[39]. Because the telomerase-null type II survivors possess long and heterogeneous telomeres, it is possible that long telomeres per se in these cells result in the life span decline. To test this possibility, yeast cells that harbored long telomeres by temporarily over-expressing telomerase were subject to a life span assay. Results show that these cells had regular life span (Figure 2E), indicating that long telomeres per se may not be the direct cause of the shorter life span we saw in the type II survivors.

To confirm that the telomerase-null type II survivors died of replicative aging but not general sickness, we examined age-dependent sterility in type II survivors. Sterility due to loss of silencing at *HMLα* and *HMRa* loci has been reported as an aging-specific phenotype in budding yeast [40],[41]. We determined the percentage of sterile cells as cells aged by documenting the inability of cells to respond to a mating pheromone, α factor. Similar to the wild-type cells, type II survivors became sterile at a higher frequency the older they got (Figure 2D). Together, the data from all the phenotypic assays in the section led us to conclude that telomerase-null type II survivors aged prematurely in a manner that was phenotypically indistinguishable from that of telomerase-positive wild-type cells.

### The life span of type II survivors can be extended by calorie restriction or by inactivation of the TOR pathway

**Figure 3:**
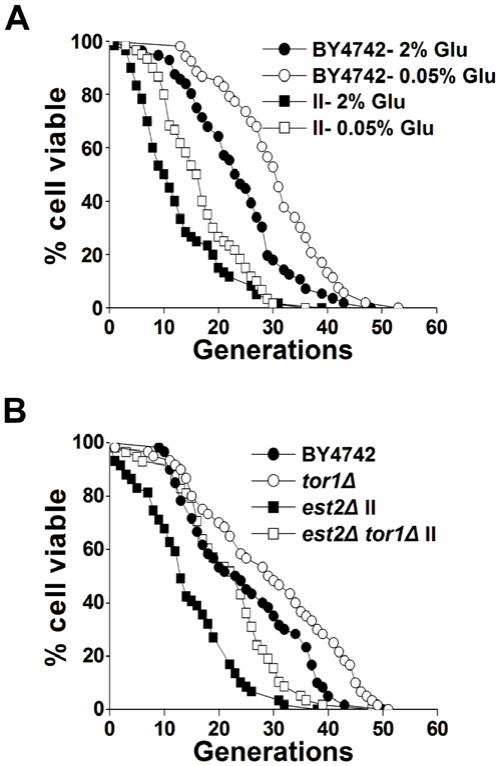
Calorie restriction or inactivation of the TOR pathway extends the life span of type II survivors. (A) Calorie restriction in *est2Δ* type II survivors. Mean life span of strains shown: BY4742-2% Glucose, 23.6±9.3 (n = 56); BY4742-0.05% Glucose, 30.5±9.2 (n = 53); *est2Δ* type II-2% Glucose, 12.6±8.0 (n = 60); *est2Δ* type II-0.05% Glucose, 16.7±7.2 (n = 60). (B) Life span analysis in *est2Δ* type II survivors with *TOR1* deletion. Mean life span of strains shown: BY4742, 24.8±11.1 (n = 60); *tor1Δ*, 29.4±12.9 (n = 60); *est2Δ* type II, 14.6±8.6 (n = 59); *tor1Δ est2Δ* type II, 22.0±9.1 (n = 58).

Calorie restriction (CR) is an intervention which slows the aging process and increases life span in many organisms [42]. In yeast, CR can be executed by reducing the glucose concentration of growth media from 2% to 0.5% (or 0.05%), resulting in a significant increase of life span (Figure 3A) [43],[44]. Life span extension by CR in yeast involves at least three nutrient-responsive kinases: TOR (target of rapamycin), Sch9, and protein kinase A (PKA) [43], [45]–[47]. To better understand life span regulation in type II survivor cells, we examined whether these canonical aging pathways function in type II survivors. Although type II survivors exhibited accelerated replicative aging, CR (in 0.05% glucose) significantly extended the survivors' life span from 12.6 to 16.7 generations (Figure 3A). The deletion of *TOR1* also significantly extended the mean and maximum life span of type II survivors (Figure 3B). Subsequently, we found that the inactivation of Tor1 affected neither the senescence nor survivor-arising rate of *est2Δ* cells (Figure S3). In addition, *tor1Δ* type II survivors exhibited shorter life span than that of *tor1Δ* telomerase-positive cells (Figure 3B). Based on these data, we propose that *TOR1* regulates the replicative life span of type II survivors independently of telomere recombination. The extension of life span by either calorie restriction or Tor1 deletion further indicated that type II survivors are not simply sick cells.

### Lack of telomerase capping does not directly cause the decline of replicative life span

**Figure 4:**
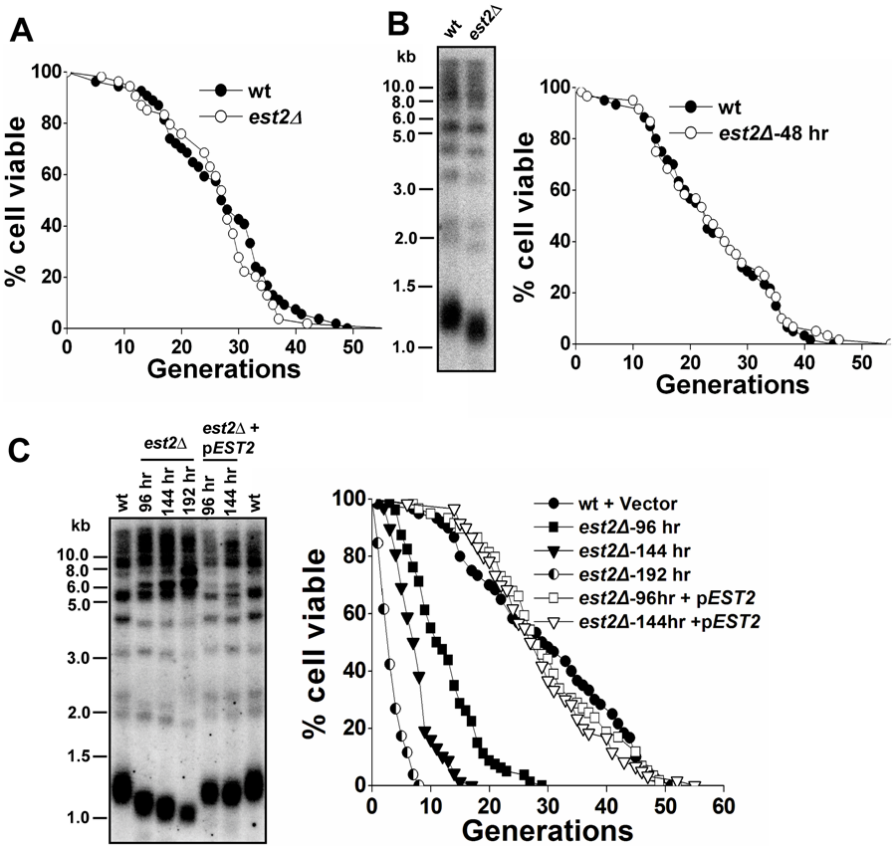
Replicative life span analysis of yeast cells with different telomere length. (A) Life span analysis of spores immediately dissected from BY4743 *EST2/est2 ::HIS3*. Average life span of strains shown (sample size): wild-type, 26.6±9.1 (n = 54); *est2Δ*, 27.0±9.9 (n = 54). (B) Life span (right) and telomere length (left) of wild-type and *est2Δ* mutants determined 48 hr growth after tetrad dissection. Average life span of strains shown (sample size): wild-type, 23.7±10.2 (n = 60); *est2Δ*, 24.3±11.0 (n = 60). (C) Life span (right) and telomere length (left) of *est2Δ* mutants determined 96, 144, or 192 hr growth after tetrad dissection; and the 96 hr, 144 hr passaged *est2Δ* cells after transformed with a functional *EST2* CEN plasmid. Average life span of strains shown (sample size): wild-type+Vector, 29.4±12.8 (n = 60); *est2Δ* -96 hr, 12.5±6.0 (n = 80); *est2Δ* -144 hr, 7.6±3.4 (n = 68); *est2Δ* -192 hr, 3.5±1.9 (n = 52); *est2Δ* -96 hr+*EST2*, 29.8±10.9 (n = 59); *est2Δ* -144 hr+*EST2*, 28.7±10.2 (n = 60).

In every way we examined them phenotypically, type II survivors seemed to resemble wild-type cells with the exception that their telomeres were elongated through telomere-telomere recombination and not telomerase (Figure 2A). Because telomerase has been suggested to play a role in capping of telomeres and facilitating cell proliferation [48], there is a possibility that the lack of telomerase capping provides an essential senescence signal. Alternatively, the increased telomere recombination per se in telomerase-null survivors induced chromosomal instability at telomere and this may be the cause of the decreased life span. To distinguish between these two possibilities, we examined the RLS of telomerase-deficient pre-survivors. Heterozygous yeast diploid cells with a single *EST2* deletion were dissected and subjected to serial restreaking on YPD plates every 48 hours. Both the spores taken immediately from the tetrad dissection and the cells that were grown on YPD plates for 48 hours had wild-type life span (Figure 4A and 4B). Decreased RLS was only observed in telomerase-null pre-survivors during the later serial restreaks when there was severe loss of telomeric DNA (Figure 4C). Reintroduction of telomerase restored the low RLS of pre-survivors by elongation of short telomeres, suggesting that the short RLS of late passages was caused by critically short telomere(s) (Figure 4C). Together, these data supported a model where the absence of Est2p did not directly cause a reduction in replicative capacity and the shorter life span of type II survivors was not caused by the lack of telomerase capping.

The observation of loss-of-productivity in late passages of telomerase-null pre-survivors mirrors what is known to happen to *est2Δ* cells on serial streak-outs (Figure 1A), or in liquid-growing culture (Figure S2A) [36],[49]. Mother cells exhibited the same rate of cell viability reduction as the logarithmically growing cells, the vast majority of which are young cells, suggesting that the older mother cells and younger cells do not have appreciable difference in their ability to maintain telomeres in the absence of telomerase.

### Reintroduction of telomerase activity restores the life span of type II survivor cells

**Figure 5:**
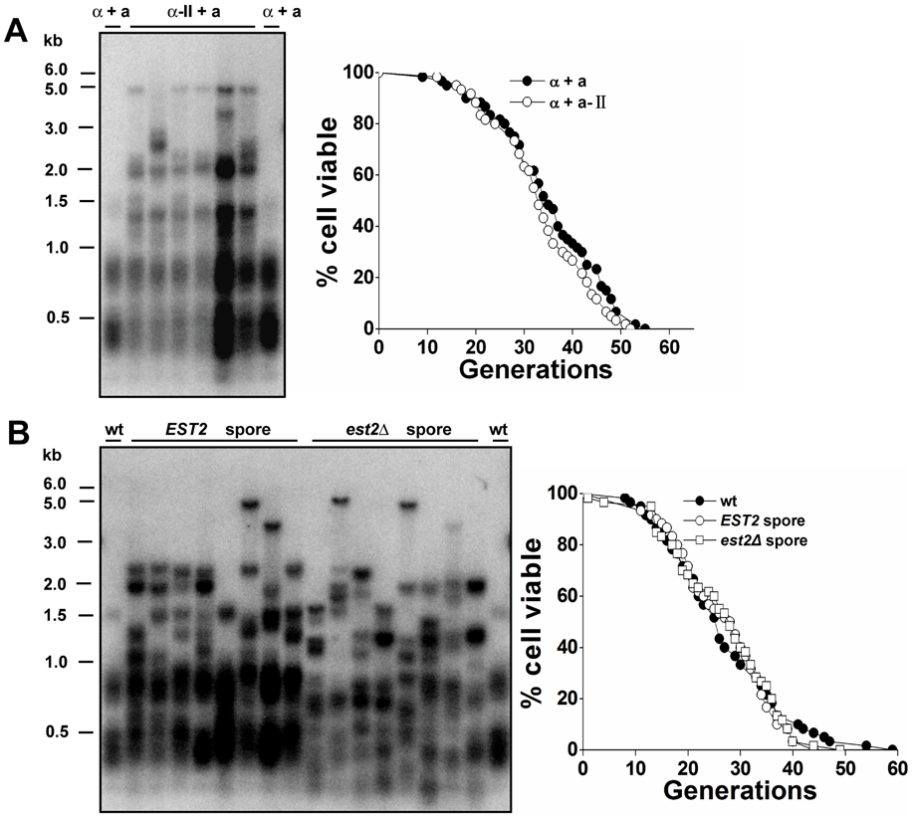
Life span measurement in type II survivors with the presence and absence of telomerase. (A) Telomere length (left) and life span (right) analysis. Diploids (named “survivor diploids”, *α*−II+*a*) were obtained from mating BY4742 *est2Δ* type II survivor (*MATα*) with BY4741 (*MATa*) wild-type cells. (*α+a*) refers to wild-type diploids from mating BY4742 (*MATα*) with BY4741 (*MATa*). (Left) Genomic DNA was digested with 4 bp cutter (*Msp*I, *Hae*III, *Hinf*I, *Alu*I). (Right) Mean life span of strains shown: *α*+*a*, 35.1±10.9 (n = 60); *α*−II+*a*, 33.5±9.6 (n = 60). (B) Telomere length (left) and life span (right) analysis. Spores were dissected from the above “survivor diploids” and their genotypes were determined. (Left) Genomic DNA was digested with 4 bp cutter (*Msp*I, *Hae*III, *Hinf*I, *Alu*I). (Right) Mean life span of strains shown: wild-type, 27.0±11.1 (n = 60); *EST2* spores, 26.8±8.9 (n = 60); *est2Δ* spores, 26.8±10.1 (n = 60).

In budding yeast, telomerase appears to be the preferred pathway for telomere maintenance [21],[22]. To examine whether reintroducing telomerase activity in survivors may inhibit telomere recombination and rescue the short RLS, we performed a mating assay in which *est2Δ* type II survivor cells (*MATα*) were mated with wild-type cells (*MATa*). The telomerase-positive diploids (named “survivor diploids”) had a heterogeneous telomere-length (Figure 5A). However, they possessed similar replicative capacity to the wild-type diploids (Figure 5A), indicating that telomerase is dominant over recombination in regulating cellular life span, and further suggesting that heterogeneous long-telomeres are not the cause of premature aging. The diploid cells in this background (BY4743) lived significantly longer than haploid cells, and this phenomenon was also observed by Kaeberlein *et al.* [50]. Next, both the telomerase-negative and positive haploid cells were obtained by tetrad dissection from the “survivor diploids”. Interestingly, all the spores had long heterogeneous telomeres but possessed the same replicative capacity as the wild-type haploids (Figure 5B). These results are supportive of the following conclusions: (i) reintroduction of telomerase activity is able to extend the short RLS of type II survivors; (ii) the reduced replicative capacity of telomerase-null type II survivors resulted from telomere alteration; (iii) lack of telomerase capping does not cause a decline of replicative capacity, consistent with the results shown in Figure 4; (iv) long telomeres per se do not affect cellular life span, consistent with our previous results shown in Figure 2E; (v) telomerase may restore RLS by inhibiting telomere recombination rather than regulating telomere length.

**Figure 6:**
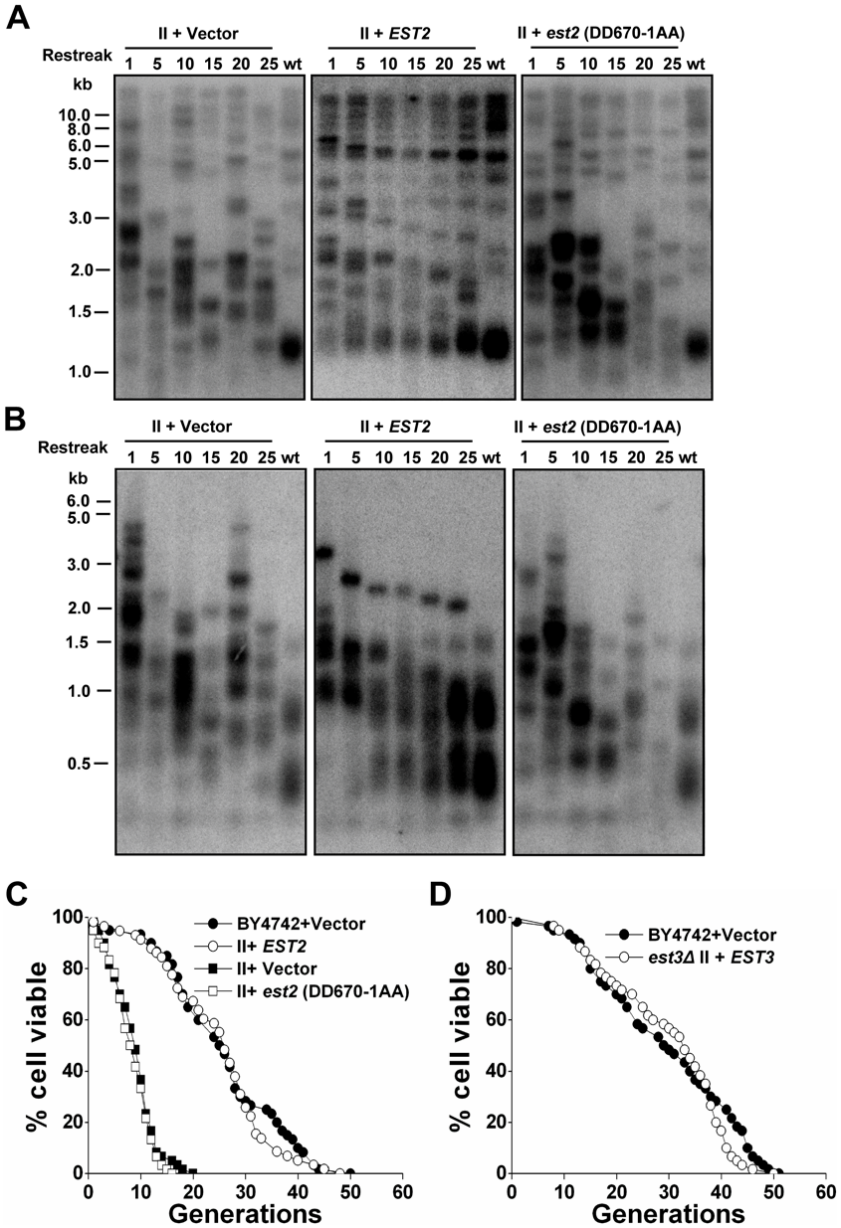
Reintroduction of telomerase activity restores telomere pattern and life span of the type II survivors. Southern blot analysis showing the dynamic changes of telomeric DNA in type II survivors after being transformed with *EST2* (wild-type), catalytically inactive *est2* (DD670-1AA), or the vector control plasmid. Cells were serially restreaked and genomic DNA from cells obtained after each indicated restreaks (labeled on top of each panel) was digested with restriction enzyme and probed with a telomere probe. (A) Genomic DNA was digested with *Xho*I. (B) Genomic DNA was digested with 4 bp cutter (*Msp*I, *Hae*III, *Hinf*I, *Alu*I). (C) Life span analysis of the 1^st^ restreaked cells after plasmid transformation. Mean life span of strains shown: BY4742+Vector, 23.3±9.5 (n = 56); *est2Δ* type II+*EST2*, 24.6±10.1 (n = 58); *est2Δ* type II+vector, 12.1±4.9 (n = 59); *est2Δ* type II+*est2* (DD670-1AA), 12.1±4.1 (n = 60). (D) Life span analysis of strains freshly transformed with *EST3* (wild-type) or the vector control plasmid. Mean life span of strains shown: BY4742+Vector, 29.4±12.8 (n = 60); *est3Δ* type II+*EST*3, 29.8±11.1 (n = 60).

To further verify the idea that reintroducing telomerase activity into survivors may inhibit telomere recombination and rescue the cellular life span, we reintroduced *EST2* into the type II survivors which were originally derived from cells with an *EST2* deletion. Reintroduction of telomerase activity caused a gradual shortening of very long telomeres in type II survivors, and eventually recovered the wild-type telomere pattern (after ∼25 restreaks, see middle panels of Figure 6A and 6B). These data suggested that when compared to recombination, telomerase was the preferred mechanism for telomere maintenance and the presence of telomerase suppressed telomere-telomere recombination [22]. Consistent with the results of our mating assay (Figure 5), the RLS of type II survivors was restored immediately after the reintroduction of *EST2* in spite of the long telomeres (Figure 6C). Accordingly, a plasmid-borne *EST3* restored the life span of *est3Δ* type II survivors immediately after being transformed (Figure 6D). Reintroduction of a catalytically inactive *est2* (DD670-1AA) into *est2Δ* type II survivors, on the other hand, failed to restore either the telomere length or the typical life span (Figure 6). These observations suggested that functional telomerase is required for the life span restoration in type II survivors.

### rDNA recombination is reduced in telomerase-null type II survivors

**Figure 7:**
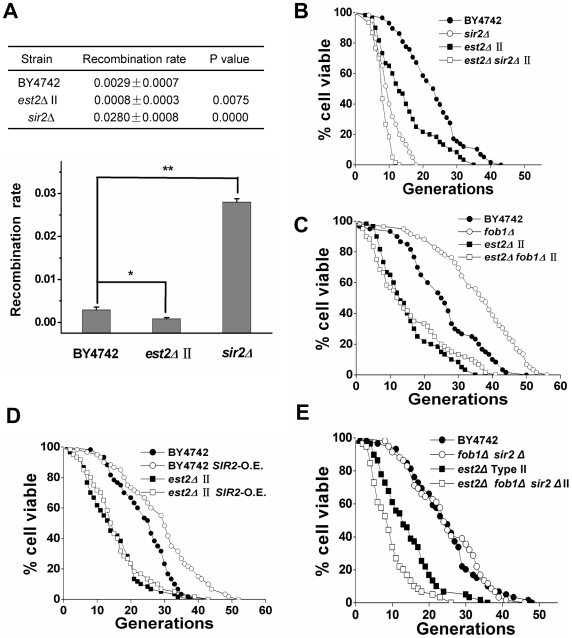
rDNA recombination is reduced in telomerase-null type II survivor cells. (A) rDNA recombination assay. The upper panel shows the average recombination rate of three independent isolates with indicated standard deviations. Approximately 20,000–30,000 colonies were examined for each strain. P values were obtained using student's t test. The lower panel contains a graph of the average recombination rates and standard deviations from the upper panel. (B) Life span analysis of *est2Δ* type II survivors with *SIR2* deletion. Mean life span of strains shown: BY4742, 23.1±8.6 (n = 58); *sir2Δ*, 10.3±3.7 (n = 60); *est2Δ* type II, 14.6±8.6 (n = 59); *sir2Δ est2Δ* type II, 8.55±4.42 (n = 53). (C) Life span analysis of *est2Δ* type II survivors with *FOB1* deletion. Mean life span of strains shown: BY4742, 25.5±10.9 (n = 60); *fob1Δ*, 35.7±12.0 (n = 59); *est2Δ* type II, 14.6±8.5 (n = 59); *fob1Δ est2Δ* type II, 13.4±10.7 (n = 59). (D) Life span analysis of *est2Δ* type II survivors with *SIR2* over-expression. Mean life span of strains shown: BY4742, 23.8±7.6 (n = 60); BY4742 *SIR2*-O.E., 29.0±11.2 (n = 60); *est2Δ* type II, 14.7±8.0 (n = 60); *est2Δ* type II *SIR2*-O.E., 15.9±8.9 (n = 60). (E) Life span analysis of *est2Δ* type II survivors with *FOB1 SIR2* double deletion. Mean life span of strains shown: BY4742, 24.3±9.7 (n = 59); *fob1Δ sir2Δ*, 24.8±9.6 (n = 59); *est2Δ* type II, 14.2±7.6 (n = 59); *fob1Δ sir2Δ est2Δ* type II, 9.6±5.6 (n = 59).

In budding yeast, a change of rDNA recombination rate causes reciprocal changes in the cellular life span. For example, elimination of the replication block protein *FOB1* or over-expression of *SIR2* significantly extended cellular life spans by reducing rDNA recombination, whereas the deletion of *SIR2* had the opposite effect [51]–[53]. In the telomerase-null type II survivors, recombination was presumably activated at the telomere loci. We wondered whether increased telomere recombination affected the stability of rDNA loci. A marker loss assay was performed as described previously [54] to analyze the rDNA recombination rate in type II survivors. Interestingly, the rDNA recombination rate in type II survivors was 3-fold lower than that observed in wild-type cells (Figure 7A), thereby suggesting that extra-chromosomal rDNA circles (ERCs) do not contribute to the acceleration of aging process in type II survivors.

Like in wild-type cells, deletion of *SIR2* in type II survivors led to a decline of life span from 14.6 to 8.6 generations (Figure 7B). Unexpectedly, neither the deletion of *FOB1* nor introduction of an extra-copy of *SIR2* into type II survivors could extend their life span (Figure 7C and 7D). Double deletion of *FOB1* and *SIR2* did not affect the life span of wild-type cells [53], whereas combined deletion of *FOB1* and *SIR2* reduced the life span of type II survivors (Figure 7E). We were unable to determine why *FOB1* deletion or Sir2 over-expression did not positively influence the RLS of type II survivors. It is possible that the increased telomere recombination sequestered recombination factors and caused a reduction in rDNA recombination (Figure 7A). Thus, either *FOB1* deletion or Sir2 over-expression could no longer reduce rDNA recombination levels that were already lower.

## Discussion

In this study, we reported that telomerase-null type II survivors, which employ homologous recombination to efficiently maintain telomeres, exhibited normal chromosomal stability in an assay that measures gross chromosomal rearrangement rates, but accelerated cellular senescence. The reduced replicative life span of type II cells could be extended by either calorie restriction or inactivation of the TOR pathway, but not by *FOB1* deletion or *SIR2* over-expression. Reintroduction of telomerase restored the life span of type II survivors to wild-type level, indicating the superiority of telomerase over homologous recombination in guaranteeing full replicative potential.

In most eukaryotic species studied so far, telomere replication involves either telomerase or a recombination pathway [9]. Stable maintenance of telomeres is required for cell proliferation, survival and preservation of a species [55]. Reactivation of telomerase or telomere-recombination is associated with immortalization of mammalian cells grown in tissue culture, including human cells [19],[56]. Similarly, the budding yeast *S. cerevisiae*, can be grown indefinitely in culture under optimal conditions with either telomerase or telomere-recombination activated for telomere maintenance (Figure 1A) [21],[22]. However, for a single yeast cell, its replicative capacity is finite due to the activity of other aging pathways regardless of how telomeres are maintained throughout the life span [33],[34]. Surprisingly, we found that the budding yeast type II survivor cells, which have adopted homologous recombination to replicate their telomeres, possessed shorter replicative life span (Figure 2 and Figure S1). Type II survivor cells differ from wild-type cells by the nature of their repetitive telomeric DNA sequences, the physiological challenges they may face, the length of their heterogeneous telomeres, the absence of telomerase capping, the heterochromatin structure at these telomeres, and the telomere recombination status. Since each of these differences alone or a combination of these differences may be responsible for the shortened RLS in type II survivors, we examined further the contributions of these differences in the premature senescence phenotype.

Compared to wild-type cells, the type II survivors did not exhibit altered sensitivity to various DNA damage-inducing reagents (Figure 1B). Additionally, they did not show an increase in the rate of gross chromosomal rearrangement events (Figure 1F). Consistent with the genetic assay, type II survivor cells and wild-type cells displayed identical chromosomal banding patterns when compared using pulsed-filed gel electrophoresis (Figure 1E). Moreover, telomere silencing was slightly enhanced in type II survivors (Figure 1C), and the telomere clustering at the nuclear periphery remained similar to the wild-type cells (Figure 1D). These results indicate that the type II survivors are phenotypically healthy, instead of generally “sick” cells.

When examining cell morphology at the end of the life span, type II survivor cells showed similar fractions of cells that were large-budded and small-budded when compared to wild-type cells at the same stage of life span (Figure 2C). In addition, the type II survivors exhibited the aging-associated sterility in a manner that was nearly identical to that of wild type cells (Figure 2D). These observations on one hand raise the argument that the type II cells are premature aging instead of premature death, and on the other hand challenge the idea that the life span reduction of type II survivors is due to their critically short telomere(s) which could cause more cells to senesce at G2/M phase (Figure S2). Additionally, the life span of type II survivors was extended by calorie restriction or inactivation of the *TOR1* pathway (Figure 3), and reduced by *SIR2* deletion (Figure 7B), further supporting the argument that the type II survivors age prematurely.

Since telomerase has been shown to play a capping function in maintaining telomere integrity [48], it remains unclear how telomere capping is maintained in the type II survivors. In the telomerase deficient pre-survivors with a moderate loss of telomeric DNA, the defect of telomerase capping due to lack of Est2p did not detectably affect the replicative capacity (Figure 4A and 4B). Consistent with those data, the telomerase-null cells newly derived from *EST2*/*est2* hybrids (crosses between type II survivors and wild-type haploids) have a comparable life span to the telomerase proficient cells regardless of the length of their heterogeneous telomeres (Figure 5B). Thus, the life span reduction in telomerase-null type II survivors did not appear to be a consequence of a loss of telomerase capping by Est2p.

Recent studies on the role of telomere length in aging have expanded from the cellular level to the anatomical/organismal level. Telomerase-deficient mice with critically short telomeres exhibit decreased viability associated with diminished proliferative capacity of B and T cells [57]–[59]. However, when telomere length is kept above the critically short length, the relationship between telomere length and life span seems to be controversial. In nematode *Caenorhabditis elegans*, Joeng *et al.* showed that worms with longer telomeres live longer [60]; whereas Raices *et al.* demonstrated that telomere length contributed little to the normal aging process [61]. In *Drosophila melanogaster*, longer telomeres were found to have no effect on the life span of the adult flies [62]. In the yeast *rif1Δ* cells, long telomeres were proposed to contribute to accelerated cellular senescence by titrating away limiting pools of Sir silencing factors from non-telomeric silenced loci [38],[39]. Several lines of evidence presented in our current study do not support the hypothesis that longer telomeres alone contribute to a shortened life span in yeast. For example, the yeast cells that harbored long telomeres by temporarily over-expressing telomerase exhibited wild-type life span (Figure 2E). Additionally, the hybrid diploid cells obtained from mating wild-type and type II haploids had full replicative capacity in spite of heterogeneous long telomeres (Figure 5A). Moreover, reintroduction of telomerase slowly restored the telomere-length homogeneity, but immediately restored the life span (Figure 6). Finally, over-expression of the essential silencing factor Sir2p had no effect on the replicative life span of type II survivors (Figure 7D). These results indicate that long-telomere length per se in the type II survivors is not associated with the accelerated cellular aging we observed. Most likely, type II survivors aged prematurely in a telomere-length independent manner. The results presented in our current work are different from the ones reported previously [38], where *rif1Δ* or *tlc1* mutants were exploited to characterize the relationship between telomere length and life span. In our experiments (Figure 2E, Figure 5, and Figure 6), no parameters other than telomere length have been changed, and this might help to explain the discrepancy of our results and those reported previously [38].

In contrast to the controversial role of telomere length in longevity determination, loss of genome integrity is generally believed to contribute to the finite life span of organisms from yeast to humans [63]. A causal link between repetitive DNA instability and aging has been previously established in *S. cerevisiae*. The rate of aging in mother cells is dictated by the stability of the rDNA, which is present in 100–150 tandem arrays of 9.1-kb repeats [33],[64],[65]. During the aging of mother cells, extra-chromosomal rDNA circles (ERCs) are formed by homologous recombination between rDNA repeats. Importantly, ERCs are self-replicating via an origin in the rDNA repeat-unit during subsequent cell cycles and they display biased segregation to mother cells due to a lack of CEN element [66]. Thus, ERCs accumulate with the aging of mother cell in a Septin- and Bud6-dependent manner, and likely contribute to cellular senescence once a threshold level is reached [67],[68]. In type II survivors, rDNA recombination was decreased compared to the wild-type cells (Figure 7A). So it is unlikely that the ERCs contributed to the acceleration of the aging process in type II survivors, and it is likely that other aging pathway(s) dominated the aging process. Accordingly, *SIR2*-overexpression or *FOB1*-deletion did not extend RLS in type II survivors because the ERC pathway was recessive in the aging process of these cells. However, *SIR2*-deletion should still further shorten RLS because it makes the ERC pathway dominant again.

As telomeres are arranged in TG-rich repeats, we could not rule out the possibility that telomere circles might affect cellular life span in the same way as rDNA circles. However, qualitative and quantitative determination of telomeric DNA-containing rings shows that telomere circles exclusively exist during the time when survivors are being generated, but not after survivors are established [69]. In addition, telomere repeats do not contain self-replicating origin elements. It's unlikely that telomere circles would accumulate during the aging process of survivors. Given that the recombination has been increased at telomeric loci in type II cells in a manner similar to that of rDNA recombination in aging cells [70], telomere recombination may titrate away vital transcription and/or replication factors that play a role in preventing cellular senescence. Accordingly, we did observe significantly reduced rDNA recombination in telomerase-null type II survivors (Figure 7A). However, we could not detect any DNA replication or repair defect on the general chromosome loci as shown by several lines of evidence. The telomerase-null type II survivors did not exhibit altered sensitivity to various DNA damage-inducing reagents when compared to wild-type cells (Figure 1B), thereby indicating there was no obvious DNA replication or repair defect on the general chromosome loci. In addition, the gross-chromosomal rearrangement (GCR) rate was not increased in type II survivors as previously reported (Figure 1F) [32]. Moreover, the chromosomal banding pattern of type II survivors was comparable to the wild-type cells as displayed by pulsed-field gel electrophoresis (Figure 1E). At this point, we could not explain why telomeres preferentially competed with the rDNA loci for recombination. One possibility is that our assays (Figure 1) are not sensitive enough to detect any replication or repair defect on the general chromosome loci. Alternatively, both telomeric and ribosomal DNAs are favorable substrates for certain factor(s), such as Sir2p binding, and an increase of recombination in either one would affect the rate of recombination at the other. However, *SIR2* over-expression, which might compensate for the decrease of Sir2p at rDNA loci, did not extend the replicative life span of type II survivors (Figure 7D), thereby leading us to propose that at least Sir2p is not the factor that might be involved in regulating the relative recombination rates at both telomeres and the rDNA.

The shorter life span of the pre-survivors, which was potentially caused by severe telomere loss, could be rescued by reintroduction of telomerase, presumably due to recovery of telomere length by telomerase (Figure 4C). Interestingly, reintroduction of telomerase immediately restored the short life span of telomerase-null type II survivors despite insignificant changes in telomere length (Figure 5 and Figure 6), implying that a distinct mechanism is engaged in the life span regulation upon reactivation of telomerase. Reintroduction of telomerase activity caused a gradual shortening of very long telomeres in type II survivors and eventually re-established the wild-type telomere Southern blotting banding pattern (Figure 6A and 6B, middle panels) as previously reported [22]. This observation suggested that the presence of telomerase somehow suppressed telomere-telomere recombination. Catalytically inactive telomerase failed to inhibit telomere recombination as reflected by the continuous presence of heterogeneous telomere pattern (Figure 6A and 6B, right panels), thus, it could not recovery the replicative life span of type II survivors (Figure 6C). We therefore propose a model where telomere recombination leads to accelerated cellular aging in telomerase-null survivors and functional telomerase rescues the replicative life span of type II survivors by inhibiting telomere recombination. In conclusion, telomerase has evolved to be as a superior mechanism to telomere recombination in regulating cellular life span. Telomerase likely plays a duel role in regulating life span. It helps maintain the telomeres above the critically short length necessary to reach full replicative potential, while also inhibiting the telomeric recombination that otherwise leads to a decline of cellular replicative capacity.

## Materials and Methods

### Yeast strains, plasmids, and media

Unless otherwise noted, all yeast strains used in this study were BY4742 (*MATα his3Δ1 leu2Δ0 lys2Δ0 ura3Δ0*), BY4741 (*MATa his3Δ1 leu2Δ0 met15Δ0 ura3Δ0*), BY4743 (*MATα/MATa his3Δ1/his3Δ1 leu2Δ0/leu2Δ0 lys2Δ0/+ met15Δ0/+ ura3Δ0/ura3Δ0*), and their derivatives. All strains were grown at 30°C and on YPD (10 g/L yeast extract, 20 g/L peptone, 2% dextrose) unless otherwise stated.

*tor1Δ*, *sir2Δ* and *fob1Δ* strains were from EUROSCARF consortium. *est2Δ* was a deletion of the *EST2* open reading frame using a pRS303 plasmid which contained 800 bp homologous sequences to up- and down-stream of *EST2* ORF. The same method was used for the deletion of the open reading frame of *EST3*. *sir2Δ fob1Δ* mutants were obtained by deletion of the *SIR2* ORF in the *fob1Δ* strain. All gene disruptions were verified by PCR. Strains over-expressing Sir2p were constructed by genomic integration of an extra-copy of *SIR2*. Integration of *SIR2* at *LEU2* locus was accomplished by transforming cells with *Hpa* I digested plasmid pRS305-*SIR2*. In addition to the entire coding region of *SIR2*, 800 nucleotides of up-stream and down-stream sequence were included. Plasmid pRS305-*SIR2* was constructed by ligation of the PCR-amplified products into the *Bam*H I and *Sal* I sites of pRS305.

The pRS316-*EST3* centromere plasmid was constructed as described [71]. The pRS316-*EST2* centromere plasmid was a gift from Dr. Yasumasa Tsukamoto. The pRS316-*est2* (DD670-1AA) was constructed using site-directed mutagenesis method.

### Yeast replicative life span analysis

Replicative life span assay of yeast cells was performed as described previously [72],[73]. Prior to analysis, strains were patched onto fresh solid medium and grown for 2 days at 30°C. Single colonies were then arrayed onto standard YPD plates using a micro-manipulator and allowed to grow for about 3 hours. Virgin daughter cells were isolated as buds from mother cells and subject to life span analysis. During life span experiments, plates were incubated at 30°C during the daytime and stored overnight (∼8 hr) at 4°C. Each experiment consisted of more than 50 mother cells and was independently repeated at least twice. Data shown in the results represent one single experiment. Statistical significance was determined by a Wilcoxon rank sum test using Stata 8 software. Differences are stated to be significant when the confidence is higher than 95%.

### Telomere blot

Genomic DNA prepared from each strain was digested by *Xho*I or 4 bp cutter (*Msp*I, *Hae*III, *Hinf*I, *Alu*I), separated on a 1.0% gel, transferred to Hybond-N+ membrane (GE Healthcare), cross-linked by UV and then probed with C_1–3_A/TG_1–3_ telomere-specific probe as described previously [22].

### Temperature and DNA damage sensitivity assay

Ten-fold dilutions of each strain were patched on YPD containing the indicated doses of phleomycin (Sigma), methyl methanesulfonate (MMS; Sigma), hydroxyurea (HU; Sigma), or exposed to UV with indicated doses, or grown at 23°C, 30°C, and 37°C. Photos were taken after two days.

### Telomere position effect analysis

Each strain that contains *URA3*-marked telomere VII-L was grown to log phase at 30°C. Ten-fold serial dilutions were plated on YC complete medium and YC containing 5-FOA (5-Fluoroorotic Acid) at 1 g/L. Plates were incubated at 30°C for two days and then photos were taken.

### Immunofluorescence

Immunostaining of Rap1 was performed as described previously [74].

### Pulsed-field gel electrophoresis

Agarose plugs for pulsed-field gel electrophoresis (PFGE) were prepared as described previously [75]. PFGE was performed on a Bio-Rad CHEF-DR-III system in 0.5×TBE at 14°C using the following program: step 1, voltage 3.6 V/cm, switch 120 s, time 20 hr; step 2, voltage 3.6 V/cm, switch 300 s, time 24 hr. After electrophoresis, DNA was visualized by ethidium bromide staining.

### Gross chromosomal rearrangement (GCR) assay

GCR rate in indicated strains was determined as previously described [76]. The nonessential gene, *HXT13*, located distal to the *CAN1*, was replaced with a second selectable marker, the *URA3* gene. Each strain was inoculated into YPD medium and grown at 30°C until the culture reached saturation. Cells of suitable dilutions were spread on YC plates in the presence or absence of 60 mg/L L-canavanine and 1 g/L 5-FOA. A fluctuation test and the method of the median were used to assess GCR rate [77].

### Determination of α factor responsiveness

Sensitivity to α factor was performed as previously described [41]. Cells of various ages were scored for their ability to undergo cell cycle arrest and schmooing in response to the yeast mating pheromone, α factor. After 4 hours of α factor challenge, cells were transferred to fresh medium to complete their life span. All cells documented underwent at least one cell division after being removed from the presence of α factor.

### Determination of rDNA recombination rate

The rate of marker loss in rDNA was measured as described [54]. Strains carrying a *URA3* marker integrated into the rDNA array were grown in YC medium lacking uracil until the culture reached saturation. Cells of suitable dilutions were spread on YC plates with and without 5-FOA. Plates were incubated at 30°C for two days and colonies were counted. The number of colonies on 5-FOA plates divided by the number of colonies on YC plates was reported as the rate of marker loss. A Student's t test was used to determine the statistical significance of the data.

## Supporting Information

Figure S1 Life span analysis of type II survivors with different genetic background. (A) Life span curves of *est2Δ* type II survivors in BY4742 (*MATα*) background. Average life span of strains shown: BY4742, 24.3±9.7 (n = 59); *est2Δ*-type II, 17.6±6.5 (n = 59). (B) Life span curves of *est2Δ* type II survivors in W303-1A (*MATa*) background. Average life span of strains shown: W303-1A, 27.2±9.4 (n = 60); *est2Δ*-type II, 15.4±6.4 (n = 59). (C) Life span curves of *est3Δ* type II survivors in BY4742 background. Average life span of strains shown: BY4742, 29.3±7.4 (n = 60); *est3Δ*-type II, 20.0±8.8 (n = 59).
(0.35 MB TIF)

Figure S2 Accumulation of G2/M-arrested cells in *est2Δ* cells. (A) The liquid viability assay of *est2Δ* mutants. Wild-type and *est2Δ* haploids were picked from a fresh dissecting plate after 48 hr at 30°C. Each colony was inoculated into 5 ml YPDA liquid culture and grown to saturation (10^8^ cells/ml) at 30°C. Serial passages were initiated with OD_600_ {similar, tilde operator} 0.05 (7.4×10^5^ cells/ml), and the cell count was measured by spectrophotometer every 24 hr. (B) FACS analysis of the percentage of G2/M-stage cells during the liquid passages. (C) Telomere blot analysis with cells from the 2^nd^, 4^th^, 8^th^, 10^th^, and 12^th^ days in liquid culture.
(1.12 MB TIF)

Figure S3 The liquid viability assay of *est2Δ* and *est2Δtor1Δ* mutants. Experimental procedure the same as Figure S2A.
(0.06 MB TIF)

## References

[1] Greider CW (1996). Telomere length regulation.. Annu Rev Biochem.

[2] Smogorzewska A, de Lange T (2004). Regulation of telomerase by telomeric proteins.. Annu Rev Biochem.

[3] Blackburn EH (2001). Switching and signaling at the telomere.. Cell.

[4] de Lange T (2002). Protection of mammalian telomeres.. Oncogene.

[5] Kass-Eisler A, Greider CW (2000). Recombination in telomere-length maintenance.. Trends Biochem Sci.

[6] Autexier C, Greider CW (1996). Telomerase and cancer: revisiting the telomere hypothesis.. Trends Biochem Sci.

[7] Zakian VA (1995). Telomeres: beginning to understand the end.. Science.

[8] Biessmann H, Mason JM (1997). Telomere maintenance without telomerase.. Chromosoma.

[9] Lundblad V (2002). Telomere maintenance without telomerase.. Oncogene.

[10] Counter CM, Meyerson M, Eaton EN, Weinberg RA (1997). The catalytic subunit of yeast telomerase.. Proc Natl Acad Sci U S A.

[11] Lingner J, Hughes TR, Shevchenko A, Mann M, Lundblad V (1997). Reverse transcriptase motifs in the catalytic subunit of telomerase.. Science.

[12] Cohn M, Edstrom JE (1992). Telomere-associated repeats in Chironomus form discrete subfamilies generated by gene conversion.. J Mol Evol.

[13] Pich U, Fuchs J, Schubert I (1996). How do Alliaceae stabilize their chromosome ends in the absence of TTTAGGG sequences?. Chromosome Res.

[14] Roth CW, Kobeski F, Walter MF, Biessmann H (1997). Chromosome end elongation by recombination in the mosquito Anopheles gambiae.. Mol Cell Biol.

[15] Mason JM, Biessmann H (1995). The unusual telomeres of Drosophila.. Trends Genet.

[16] Biessmann H, Mason JM, Ferry K, d'Hulst M, Valgeirsdottir K (1990). Addition of telomere-associated HeT DNA sequences “heals” broken chromosome ends in Drosophila.. Cell.

[17] Danilevskaya O, Slot F, Pavlova M, Pardue ML (1994). Structure of the Drosophila HeT-A transposon: a retrotransposon-like element forming telomeres.. Chromosoma.

[18] Traverse KL, Pardue ML (1988). A spontaneously opened ring chromosome of Drosophila melanogaster has acquired He-T DNA sequences at both new telomeres.. Proc Natl Acad Sci U S A.

[19] Kim NW, Piatyszek MA, Prowse KR, Harley CB, West MD (1994). Specific association of human telomerase activity with immortal cells and cancer.. Science.

[20] Bryan TM, Englezou A, Dalla-Pozza L, Dunham MA, Reddel RR (1997). Evidence for an alternative mechanism for maintaining telomere length in human tumors and tumor-derived cell lines.. Nat Med.

[21] Lundblad V, Blackburn EH (1993). An alternative pathway for yeast telomere maintenance rescues est1- senescence.. Cell.

[22] Teng SC, Zakian VA (1999). Telomere-telomere recombination is an efficient bypass pathway for telomere maintenance in Saccharomyces cerevisiae.. Mol Cell Biol.

[23] Dunham MA, Neumann AA, Fasching CL, Reddel RR (2000). Telomere maintenance by recombination in human cells.. Nat Genet.

[24] Yeager TR, Neumann AA, Englezou A, Huschtscha LI, Noble JR (1999). Telomerase-negative immortalized human cells contain a novel type of promyelocytic leukemia (PML) body.. Cancer Res.

[25] Chen Q, Ijpma A, Greider CW (2001). Two survivor pathways that allow growth in the absence of telomerase are generated by distinct telomere recombination events.. Mol Cell Biol.

[26] Liti G, Louis EJ (2003). NEJ1 prevents NHEJ-dependent telomere fusions in yeast without telomerase.. Mol Cell.

[27] Mortimer RK, Johnston JR (1959). Life span of individual yeast cells.. Nature.

[28] Makovets S, Williams TL, Blackburn EH (2008). The telotype defines the telomere state in Saccharomyces cerevisiae and is inherited as a dominant non-Mendelian characteristic in cells lacking telomerase.. Genetics.

[29] Eugster A, Lanzuolo C, Bonneton M, Luciano P, Pollice A (2006). The finger subdomain of yeast telomerase cooperates with Pif1p to limit telomere elongation.. Nat Struct Mol Biol.

[30] Kyrion G, Liu K, Liu C, Lustig AJ (1993). RAP1 and telomere structure regulate telomere position effects in Saccharomyces cerevisiae.. Genes Dev.

[31] Renauld H, Aparicio OM, Zierath PD, Billington BL, Chhablani SK (1993). Silent domains are assembled continuously from the telomere and are defined by promoter distance and strength, and by SIR3 dosage.. Genes Dev.

[32] Pennaneach V, Kolodner RD (2004). Recombination and the Tel1 and Mec1 checkpoints differentially effect genome rearrangements driven by telomere dysfunction in yeast.. Nat Genet.

[33] Sinclair DA, Guarente L (1997). Extrachromosomal rDNA circles–a cause of aging in yeast.. Cell.

[34] D'Mello NP, Jazwinski SM (1991). Telomere length constancy during aging of Saccharomyces cerevisiae.. J Bacteriol.

[35] Jazwinski SM (1990). Aging and senescence of the budding yeast Saccharomyces cerevisiae.. Mol Microbiol.

[36] AS IJ, Greider CW (2003). Short telomeres induce a DNA damage response in Saccharomyces cerevisiae.. Mol Biol Cell.

[37] Johnson FB, Marciniak RA, McVey M, Stewart SA, Hahn WC (2001). The Saccharomyces cerevisiae WRN homolog Sgs1p participates in telomere maintenance in cells lacking telomerase.. Embo J.

[38] Austriaco NR, Guarente LP (1997). Changes of telomere length cause reciprocal changes in the lifespan of mother cells in Saccharomyces cerevisiae.. Proc Natl Acad Sci U S A.

[39] Kennedy BK, Gotta M, Sinclair DA, Mills K, McNabb DS (1997). Redistribution of silencing proteins from telomeres to the nucleolus is associated with extension of life span in S. cerevisiae.. Cell.

[40] Sinclair DA, Mills K, Guarente L (1997). Accelerated aging and nucleolar fragmentation in yeast sgs1 mutants.. Science.

[41] Smeal T, Claus J, Kennedy B, Cole F, Guarente L (1996). Loss of transcriptional silencing causes sterility in old mother cells of S. cerevisiae.. Cell.

[42] Mair W, Dillin A (2008). Aging and survival: the genetics of life span extension by dietary restriction.. Annu Rev Biochem.

[43] Lin SJ, Defossez PA, Guarente L (2000). Requirement of NAD and SIR2 for life-span extension by calorie restriction in Saccharomyces cerevisiae.. Science.

[44] Tsuchiya M, Dang N, Kerr EO, Hu D, Steffen KK (2006). Sirtuin-independent effects of nicotinamide on lifespan extension from calorie restriction in yeast.. Aging Cell.

[45] Fabrizio P, Pozza F, Pletcher SD, Gendron CM, Longo VD (2001). Regulation of longevity and stress resistance by Sch9 in yeast.. Science.

[46] Kaeberlein M, McDonagh T, Heltweg B, Hixon J, Westman EA (2005). Substrate-specific activation of sirtuins by resveratrol.. J Biol Chem.

[47] Powers RW, Kaeberlein M, Caldwell SD, Kennedy BK, Fields S (2006). Extension of chronological life span in yeast by decreased TOR pathway signaling.. Genes Dev.

[48] Chan SW, Blackburn EH (2002). New ways not to make ends meet: telomerase, DNA damage proteins and heterochromatin.. Oncogene.

[49] Lendvay TS, Morris DK, Sah J, Balasubramanian B, Lundblad V (1996). Senescence mutants of Saccharomyces cerevisiae with a defect in telomere replication identify three additional EST genes.. Genetics.

[50] Kaeberlein M, Kirkland KT, Fields S, Kennedy BK (2005). Genes determining yeast replicative life span in a long-lived genetic background.. Mech Ageing Dev.

[51] Park PU, Defossez PA, Guarente L (1999). Effects of mutations in DNA repair genes on formation of ribosomal DNA circles and life span in Saccharomyces cerevisiae.. Mol Cell Biol.

[52] Defossez PA, Prusty R, Kaeberlein M, Lin SJ, Ferrigno P (1999). Elimination of replication block protein Fob1 extends the life span of yeast mother cells.. Mol Cell.

[53] Kaeberlein M, McVey M, Guarente L (1999). The SIR2/3/4 complex and SIR2 alone promote longevity in Saccharomyces cerevisiae by two different mechanisms.. Genes Dev.

[54] Kobayashi T, Horiuchi T, Tongaonkar P, Vu L, Nomura M (2004). SIR2 regulates recombination between different rDNA repeats, but not recombination within individual rRNA genes in yeast.. Cell.

[55] Blasco MA (2007). Telomere length, stem cells and aging.. Nat Chem Biol.

[56] Henson JD, Neumann AA, Yeager TR, Reddel RR (2002). Alternative lengthening of telomeres in mammalian cells.. Oncogene.

[57] Blasco MA, Lee HW, Hande MP, Samper E, Lansdorp PM (1997). Telomere shortening and tumor formation by mouse cells lacking telomerase RNA.. Cell.

[58] Lee HW, Blasco MA, Gottlieb GJ, Horner JW, Greider CW (1998). Essential role of mouse telomerase in highly proliferative organs.. Nature.

[59] Herrera E, Samper E, Martin-Caballero J, Flores JM, Lee HW (1999). Disease states associated with telomerase deficiency appear earlier in mice with short telomeres.. Embo J.

[60] Joeng KS, Song EJ, Lee KJ, Lee J (2004). Long lifespan in worms with long telomeric DNA.. Nat Genet.

[61] Raices M, Maruyama H, Dillin A, Karlseder J (2005). Uncoupling of longevity and telomere length in C. elegans.. PLoS Genet.

[62] Walter MF, Biessmann MR, Benitez C, Torok T, Mason JM (2007). Effects of telomere length in Drosophila melanogaster on life span, fecundity, and fertility.. Chromosoma.

[63] Burhans WC, Weinberger M (2007). DNA replication stress, genome instability and aging.. Nucleic Acids Res.

[64] Philippsen P, Thomas M, Kramer RA, Davis RW (1978). Unique arrangement of coding sequences for 5 S, 5.8 S, 18 S and 25 S ribosomal RNA in Saccharomyces cerevisiae as determined by R-loop and hybridization analysis.. J Mol Biol.

[65] Rustchenko EP, Sherman F (1994). Physical constitution of ribosomal genes in common strains of Saccharomyces cerevisiae.. Yeast.

[66] Murray AW, Szostak JW (1983). Pedigree analysis of plasmid segregation in yeast.. Cell.

[67] Shcheprova Z, Baldi S, Frei SB, Gonnet G, Barral Y (2008). A mechanism for asymmetric segregation of age during yeast budding.. Nature.

[68] Johnson FB, Sinclair DA, Guarente L (1999). Molecular biology of aging.. Cell.

[69] Lin CY, Chang HH, Wu KJ, Tseng SF, Lin CC (2005). Extrachromosomal telomeric circles contribute to Rad52-, Rad50-, and polymerase delta-mediated telomere-telomere recombination in Saccharomyces cerevisiae.. Eukaryot Cell.

[70] Sinclair DA, Mills K, Guarente L (1998). Molecular mechanisms of yeast aging.. Trends Biochem Sci.

[71] Yang CP, Chen YB, Meng FL, Zhou JQ (2006). Saccharomyces cerevisiae Est3p dimerizes in vitro and dimerization contributes to efficient telomere replication in vivo.. Nucleic Acids Res.

[72] Kaeberlein M, Kirkland KT, Fields S, Kennedy BK (2004). Sir2-independent life span extension by calorie restriction in yeast.. PLoS Biol.

[73] Fu XH, Meng FL, Hu Y, Zhou JQ (2008). Candida albicans, a distinctive fungal model for cellular aging study.. Aging Cell.

[74] Gotta M, Laroche T, Formenton A, Maillet L, Scherthan H (1996). The clustering of telomeres and colocalization with Rap1, Sir3, and Sir4 proteins in wild-type Saccharomyces cerevisiae.. J Cell Biol.

[75] Maringele L, Lydall D, Xiao W (2006). Pulsed-Field Gel Electrophoresis of Budding Yeast Chromosomes.. Methods in Molecular Biology: Yeast Protocols.

[76] Chen C, Kolodner RD (1999). Gross chromosomal rearrangements in Saccharomyces cerevisiae replication and recombination defective mutants.. Nat Genet.

[77] Lea DE, Coulson CA (1949). The distribution of the number of mutants in bacterial populations.. J Genet.

