# Supplementary figures and images for: Telomere Recombination Accelerates Cellular Aging in Saccharomyces cerevisiae
Source: PLoS Genet. 2009 Jun 26;5(6):e1000535. doi: 10.1371/journal.pgen.1000535 (PMC2694356; doi:10.1371/journal.pgen.1000535)

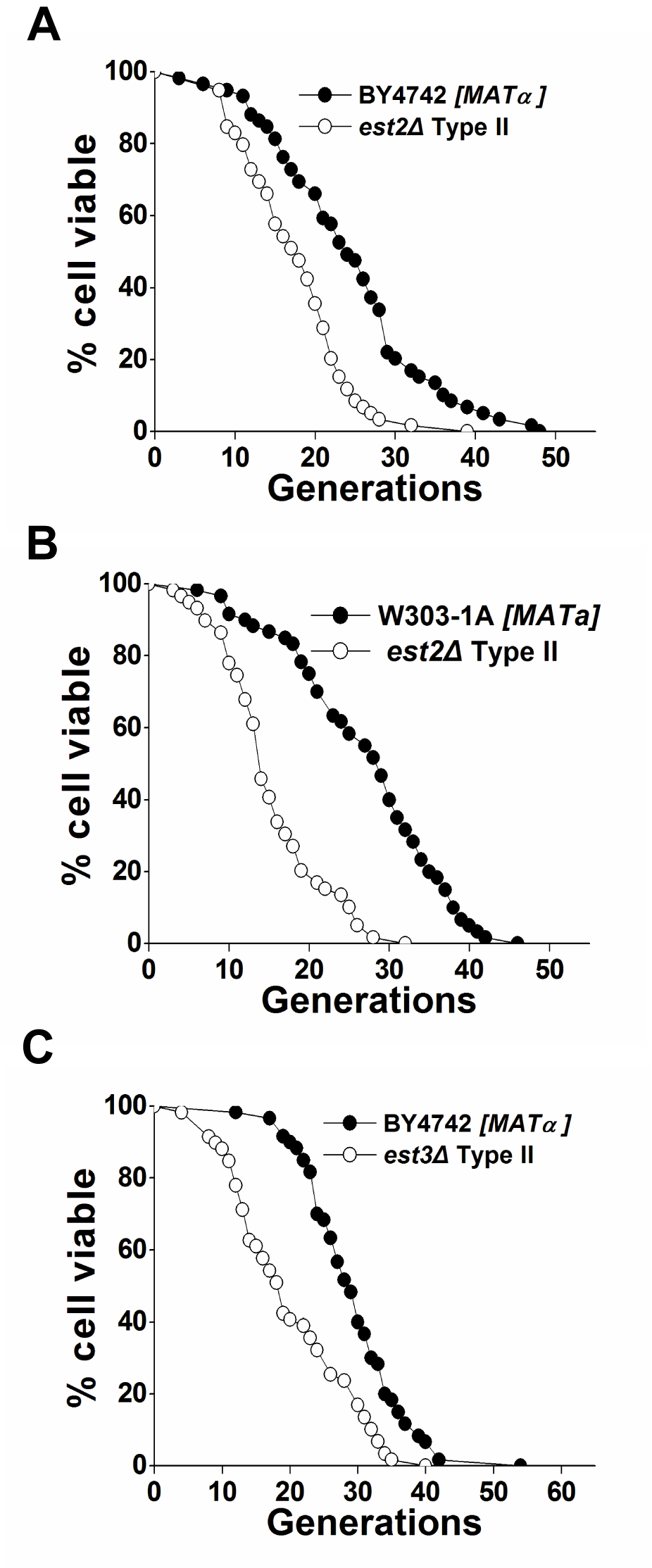

Supplement: Figure S1 — Life span analysis of type II survivors with different genetic background. (A) Life span curves of est2Δ type II survivors in BY4742 (MATα) background. Average life span of strains shown: BY4742, 24.3±9.7 (n = 59); est2Δ-type II, 17.6±6.5 (n = 59). (B) Life span curves of est2Δ type II survivors in W303-1A (MATa) background. Average life span of strains shown: W303-1A, 27.2±9.4 (n = 60); est2Δ-type II, 15.4±6.4 (n = 59). (C) Life span curves of est3Δ type II survivors in BY4742 background. Average life span of strains shown: BY4742, 29.3±7.4 (n = 60); est3Δ-type II, 20.0±8.8 (n = 59). (0.35 MB TIF) [file pgen.1000535.s001.tif]

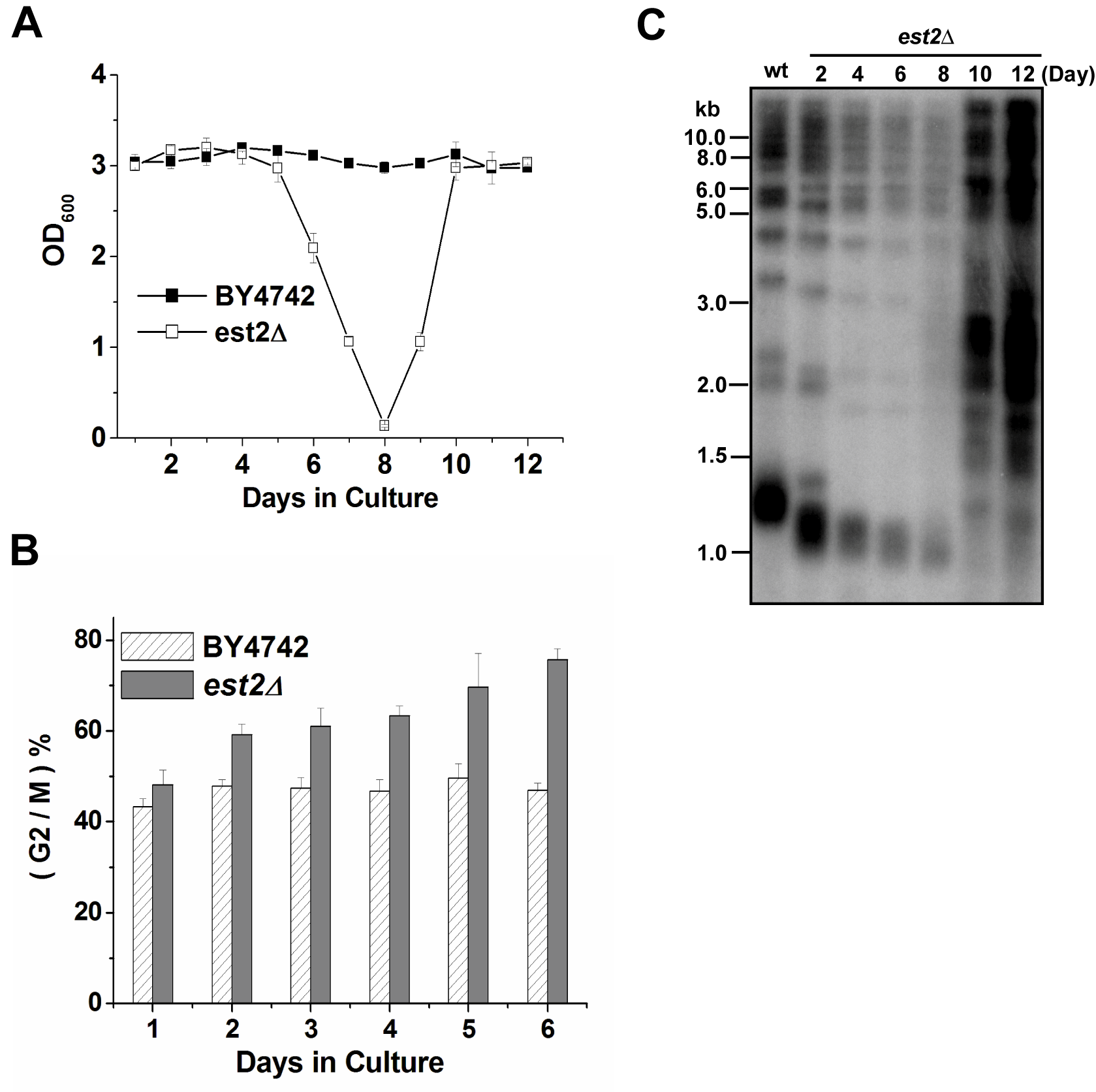

Supplement: Figure S2 — Accumulation of G2/M-arrested cells in est2Δ cells. (A) The liquid viability assay of est2Δ mutants. Wild-type and est2Δ haploids were picked from a fresh dissecting plate after 48 hr at 30°C. Each colony was inoculated into 5 ml YPDA liquid culture and grown to saturation (108 cells/ml) at 30°C. Serial passages were initiated with OD600 {similar, tilde operator} 0.05 (7.4×10^5^ cells/ml), and the cell count was measured by spectrophotometer every 24 hr. (B) FACS analysis of the percentage of G2/M-stage cells during the liquid passages. (C) Telomere blot analysis with cells from the 2nd, 4th, 8th, 10th, and 12th days in liquid culture. (1.12 MB TIF) [file pgen.1000535.s002.tif]

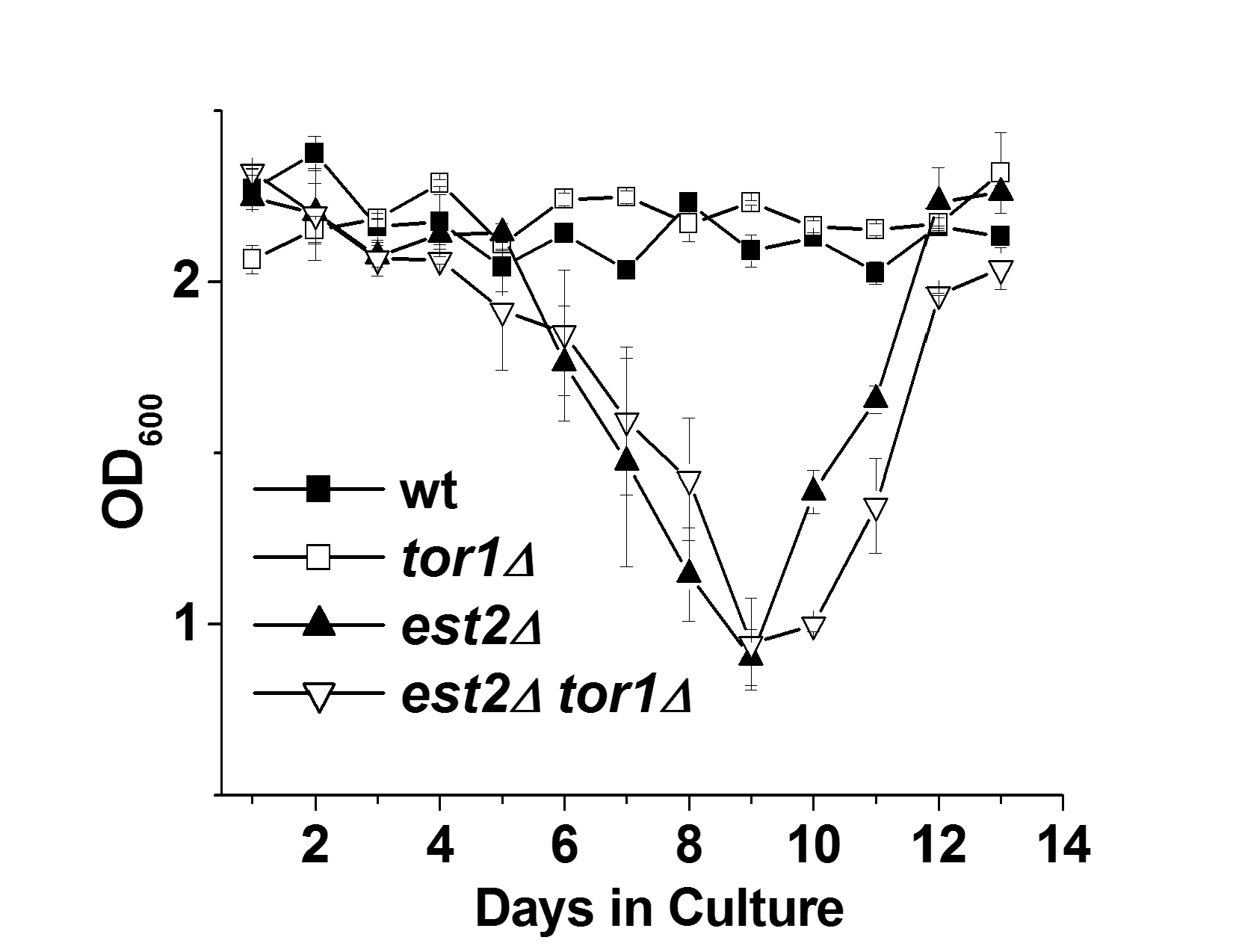

Supplement: Figure S3 — The liquid viability assay of est2Δ and est2Δtor1Δ mutants. Experimental procedure the same as Figure S2A. (0.06 MB TIF) [file pgen.1000535.s003.tif]
